# *Cupressus arizonica* Greene: Phytochemical Profile and Cosmeceutical and Dermatological Properties of Its Leaf Extracts

**DOI:** 10.3390/molecules28031036

**Published:** 2023-01-20

**Authors:** Nora Tawfeek, Eman Fikry, Ismail Mahdi, Melvin Adhiambo Ochieng, Widad Ben Bakrim, Noamane Taarji, Mona F. Mahmoud, Mansour Sobeh

**Affiliations:** 1 Department of Pharmacognosy, Faculty of Pharmacy, Zagazig University, Zagazig 44519, Egypt; 2 AgroBioSciences, Mohammed VI Polytechnic University, Lot 660–Hay MoulayRachid, Ben-Guerir 43150, Morocco; 3 African Sustainable Agriculture Research Institute (ASARI), Mohammed VI Polytechnic University (UM6P), Laayoune 70022, Morocco; 4 Department of Pharmacology and Toxicology, Faculty of Pharmacy, Zagazig University, Zagazig 44519, Egypt

**Keywords:** *Cupressus arizonica* Greene, cosmeceutical, collagenase, elastase, tyrosinase, hyaluronidase, biofilm inhibition, dermatological

## Abstract

For many decades, natural resources have traditionally been employed in skin care. Here, we explored the phytochemical profile of the aqueous and ethanolic leaf extracts of *Cupressus arizonica* Greene and assessed their antioxidant, antiaging and antibacterial activities in vitro. Liquid chromatography–mass spectrometry (LC-MS/MS) analysis led to the tentative identification of 67 compounds consisting mainly of phenolic and fatty acids, diterpene acids, proanthocyanidins and flavonoid and biflavonoid glycosides. The aqueous extract demonstrated substantial in vitro antioxidant potential at FRAP and DPPH assays and inhibited the four target enzymes (collagenase, elastase, tyrosinase, and hyaluronidase) engaged in skin remodeling and aging with IC_50_ values close to those of the standard drugs. Moreover, the aqueous extract at 25 mg/mL suppressed biofilm formation by *Pseudomonas aeruginosa*, a bacterial pathogen causing common skin manifestations, and decreased its swarming and swimming motilities. In conclusion, *C. arizonica* leaves can be considered a promising candidate for potential application in skin aging.

## 1. Introduction

Skin represents the largest and most complex part of our body. Skin aging is a complicated biological phenomenon associated with alterations in the physical, structural, and physiological characters of the epidermis and dermis. During aging, the skin suffers from several adverse visible signs including dryness, wrinkle formation, reduction in skin elasticity, decreased wound healing capacity, uneven pigmentation, and discoloration. Skin aging is prompted by several extrinsic and intrinsic factors. The latter include hormonal, genetic, and cellular metabolic factors, while extrinsic factors include chronic exposure to external agents such as chemicals, pollutants, toxins, and predominantly, exposure to sunlight ultraviolet radiation, particularly UV-B, which leads to photoaging and potentially skin cancer. These factors ultimately lead to free radicals or reactive oxygen species (ROS), the generation of which further triggers the phenomenon of oxidative stress (OxS) in the skin [1].

Free radicals or ROS potentially stimulate the oxidation of skin lipids and proteins, leading to DNA damage and cell death that elicit a change in structural skin cell components and destruction of the cell membranes. In addition, an increase in matrix metalloproteinases (MMPs) expression in human skin can be enhanced by excessive ROS and OxS. Such proteolytic enzymes (MMPs) degrade extracellular matrix (ECM) proteins including elastin, collagen and hyaluronic acid [1]. The breakdown of collagen network and elastin fibers by MMP family members collagenase and elastase results in the loss of skin elasticity and appearance of wrinkles and consequently promotes skin aging. Additionally, hyaluronic acid, which is necessary to maintain the moisture of the skin as well as its smoothness and plasticity, is subjected to cleavage by the hyaluronidase, leading to inadequate skin hydration with subsequent dryness and sagging. Furthermore, one of the notable signs of skin aging is the excessive production of melanin, which is regulated by the tyrosinase enzyme. Although melanin has a vital role in the skin against UV-induced damage, its overproduction causes various skin problems such as hyperpigmentation and age spots. Therefore, these previously mentioned enzymes can be considered as potential targets of phytochemical inhibitors, especially those with significant antioxidant capacity, against skin aging and hyperpigmentation [2,3].

Biofilms are complex microbial cells embedded in an extracellular matrix composed of polysaccharides, proteins, extracellular DNA, and lipids. These biofilms provide an extra covering around their aggregated cells; thus, bacteria within the biofilm are more resistant to different groups of antimicrobial agents and even escape host immune reactions, causing many health problems [4]. The reduced sensitivity of biofilm-forming bacteria to antibiotics may be due to the fact that the surface layer of a biofilm is only exposed to lethal levels of antibiotics because of deactivation, adsorption of antibiotics to the biofilm extracellular matrix or other biochemical, physical or mechanical mechanisms that limit the effective penetration of antibiotics into the biofilm substratum [5]. *Pseudomonas aeruginosa* is a Gram-negative opportunistic bacterium and one of the pathogenic biofilm-producing bacteria that can cause different infections related to the skin. Recently, it has been the focus of intense research owing to its well-known involvement in diseases and increased resistance to antimicrobial agents [6,7]. Various plant extracts have been reported to inhibit *P. aeruginosa* biofilm production [8,9,10]. Currently, the discovery of natural resources for cosmeceutical and dermatological agents has become a crucial aim to meet consumers’ requirements and avoid the adverse effects of synthetic ingredients [2].

Gymnosperms possess the potential to supply promising candidates for drug discovery, but comprehensive studies have not investigated many plants. Conifers are a class of cone-bearing trees and shrubs belonging to the gymnosperms. The Cupressaceae family is considered the third largest gymnosperm family and one of the most important conifers. It includes 28–33 genera and 130–136 species of evergreen monoecious or dioecious trees and shrubs. Cupressaceae members have ornamental, economic and medicinal importance [11,12].

The genus *Cupressus* (cypress) is the second largest genus belonging to the family Cupressaceae. It comprises about 25 species of monoecious trees or large shrubs. It is mostly distributed in the northern hemisphere, including Asia, the Mediterranean region, northwest Africa, southern China, and North and Central America. The leaves are opposite decussate, and needle-like on young plants (up to 2 years old) and scale-like on adult trees. The seed cones are terminal and woody, maturing 1–2 years from pollination. Pollen cones are usually terminal and yellow. The seeds are many per scale, small, winged, and flat to angled in shape. *Cupressus* species have ornamental value because of their pyramidal or columnar crown shape and were used traditionally for the treatment of rheumatoid arthritis, pertussis, and respiratory problems. *Cupressus* members are well known to produce both volatile (essential oils) and nonvolatile constituents, mainly flavonoids, biflavonoids and diterpenes [12,13,14,15].

*Cupressus arizonica* Greene (Arizona cypress) is an evergreen tree (5–25 m in height). Stems are short, 1–2 mm in diameter and four-sided. The leaves are dull greyish green to bright blue green, scale-like, and bear rounded shoots. Pollen cones are 2–5 mm in length, 2–2.5 mm in diameter, and cylindric to four-sided in shape, while seed cones are 10–35 mm in length, spherical to ovoid, and open after maturation. *C. arizonica* is widely distributed in the southwestern United States and Mexico [13,16].

*C. arizonica* leaves are rich in volatile oils [17,18], which showed insecticidal [18], antifungal [19] as well as larvicidal activities [20]. To the best of our knowledge, so far, there has been no comprehensive phytochemical investigation on biflavonoids, flavonoids, other phenolic compounds and diterpenoids of the leaves, which motivated us to investigate a comprehensive map of the aqueous and ethanolic leaf extracts using HPLC-MS/MS. This study was also intended to confirm the antibiofilm activity of *C. arizonica* leaf extracts against a strong biofilm-forming bacterium, *Pseudomonas aeruginosa*, and investigate the in vitro antioxidant and inhibitory activities of the extracts against the different skin key enzymes, mainly elastase, hyaluronidase, collagenase and tyrosinase.

## 2. Results

### 2.1. In Vitro Antioxidant Assays

The in vitro antioxidant activities of the aqueous and ethanolic extracts of *C. arizonica* leaves using DPPH and FRAP assays are presented in Table 1. Results of the present study showed that the aqueous extract had higher total phenolic (TPC) and total flavonoid (TFC) contents than the ethanolic extract. The TPC ranged from 139.99 ± 0.04 to 88.86 ± 0.08 GA/g extract, and the TFC ranged from 1.54 ± 0.05 to 1.12 ± 0.16 mg quercetin/g extract, respectively (Table 1). In accordance with this finding, the aqueous extract displayed conspicuous antioxidant activity, exhibiting an IC_50_ of 55.45 ± 0.51 μg/mL in DPPH and 15.1 ± 0.04 mM FeSO_4_ equivalent/mg sample in FRAP assay, in comparison to quercetin and BHT, respectively (Table 1), whereas the ethanolic extract showed an IC_50_ of 61.89 ± 0.11 μg/mL in DPPH and 11.56 ± 0.01 mM FeSO_4_ equivalent/mg sample in FRAP assay, in comparison to quercetin and BHT, respectively (Table 1). Similar results were also described for the ethanolic extract of wood knots from Iran [21].

### 2.2. In Vitro Anti-Aging Activities

The two extracts were investigated for their in vitro blocking activity against four enzymes involved in skin aging, namely elastase, tyrosinase, collagenase and hyaluronidase (Table 2). The IC_50_ of aqueous extract that inhibited these enzymes was much less than that of the ethanolic extract. It exhibited stronger inhibition against tyrosinase, elastase, and hyaluronidase, respectively, and showed less inhibition against collagenase compared to the references kojic acid and quercetin.

### 2.3. Antibacterial Activities

#### 2.3.1. Minimum Inhibitory Concentration

**Figure 1:**
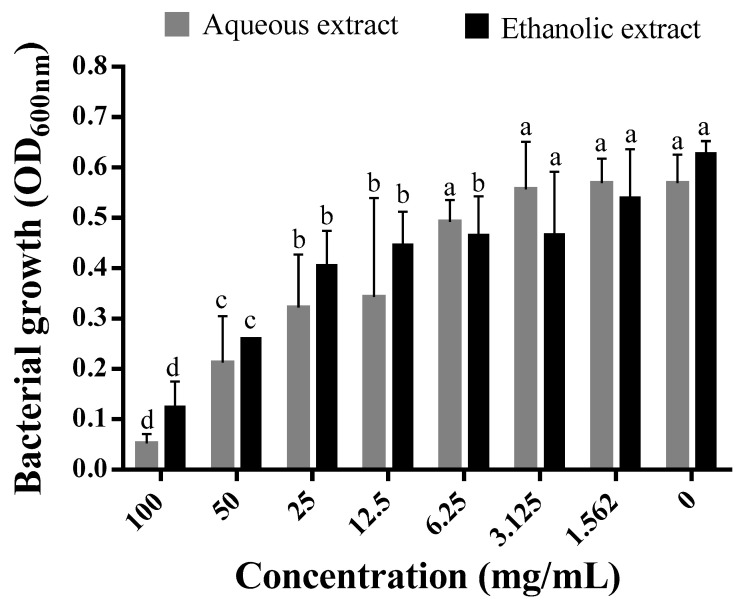
Effect of the different concentrations of aqueous and ethanolic extracts of *C. arizonica* leaves on bacterial growth rate of *P. aeruginosa*. Different letters in superscript indicate a statistical difference at *p* < 0.05.

The growth of *P. aeruginosa* in MH media supplemented with increasing concentrations of each extract was inhibited in a dose-dependent manner by the extract (Figure 1). A significant decrease in the turbidity of the suspension caused by bacterial growth was observed starting from 12.5 mg/mL of both extracts. Comparatively, the bacterial growth in media amended with the aqueous extract was lower than in media containing the ethanolic extract. Notably, the MIC for both extracts was >100 mg/mL, as there was no complete inhibition at the highest concentration tested. Nevertheless, growth was inhibited by 79.83 and 90.71% using the aqueous and ethanolic extracts, respectively (Figure 1). Using the standard antibiotic, ampicillin, the complete inhibition of *P. aeruginosa* growth was noted at 0.3125 mg/mL.

#### 2.3.2. Effect of *C. arizonica* Leaf Extracts on Biofilm Production

**Figure 2:**
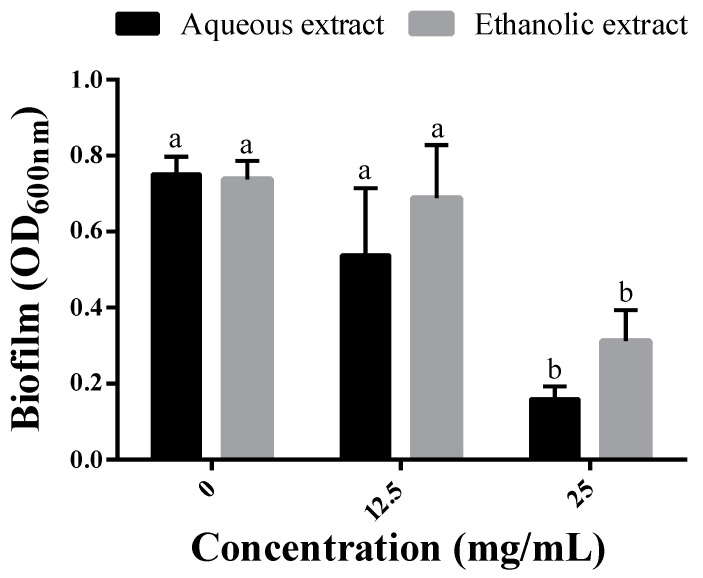
Biofilm production by *P. aeruginosa* grown in the absence (0 mg/mL) and presence of the extracts at 12.5 mg/mL (1/8 MIC) and 25 mg/mL (1/4 MIC). Different letters in superscript indicate a statistical difference at *p* < 0.05.

The biofilm production by *P. aeruginosa* in MH media supplemented with 1/8 and 1/4 MICs of each extract was estimated using the crystal violet assay. At 1/8 MIC, corresponding to 12.5 mg/mL for both extracts, no significant decreases were noted as compared to extract-free MH media. However, when the dose was increased to 1/4 MIC (25 mg/mL), the amount of produced biofilm was significantly reduced by the two extracts. These decreases reached 78.8 and 57.9% using the aqueous and ethanolic extracts, respectively (Figure 2).

#### 2.3.3. Effect of *C. arizonica* Leaf Extracts on the Swimming and Swarming Motilities

**Figure 3:**
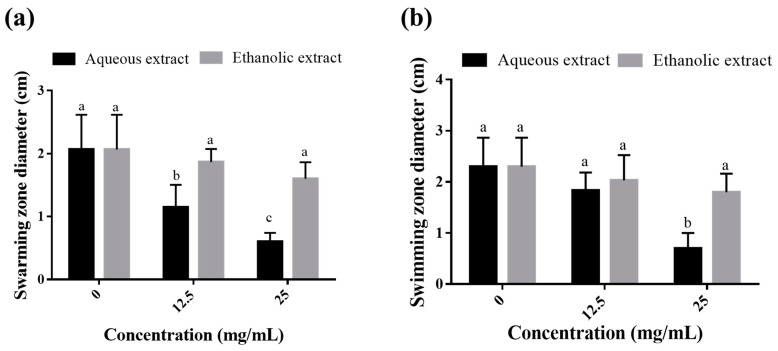
Effect of sub-MICs of *C. arizonica* leaf extracts on the swarming (**a**) and swimming (**b**) motilities of *P. aeruginosa* on agar plates. Different letters in superscript indicate a statistical difference at *p* < 0.05.

The swarming and swimming motilities of *P. aeruginosa* on agar plates supplemented with 1/8 and 1/4 MICs were monitored as they are crucial mechanisms allowing pathogens to move across surfaces during infections. As shown in Figure 3a, the aqueous extract was more potent than the ethanolic extract in inhibiting the swarming motility of the bacterium (in a dose-dependent manner by up to 70.8%). However, the ethanolic extract showed no significant effect at the two concentrations tested. As for the swimming assay, the aqueous extract was active at only 1/4 MIC (25 mg/mL) with 69.56% inhibition, while the ethanolic extract exhibited no inhibitory effect on the swimming ability of the tested bacterium (Figure 3b).

### 2.4. Phytochemical Profiling 

**Figure 4:**
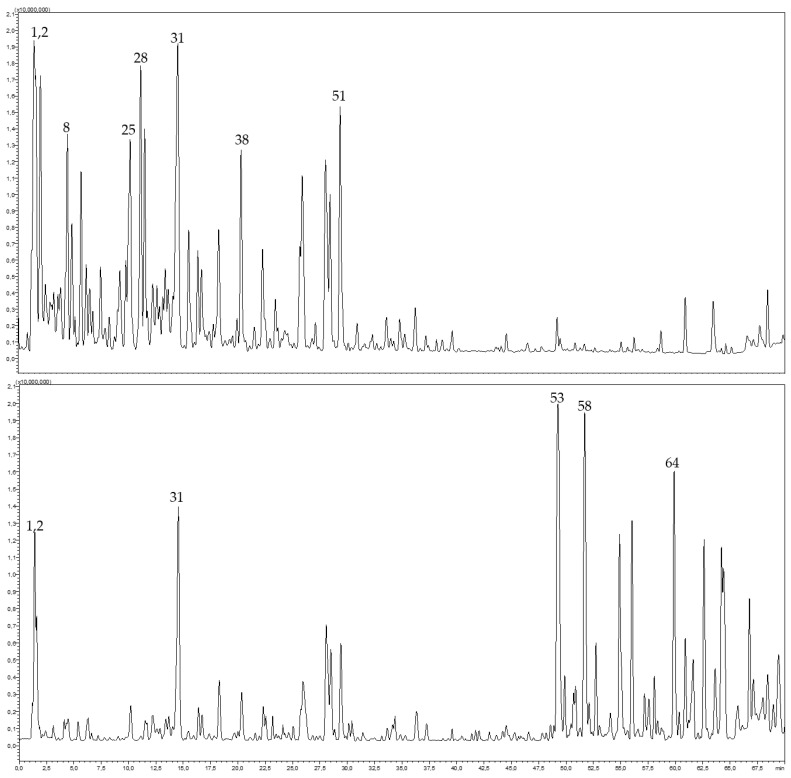
HPLC-MS profile of aqueous extract (top) and ethanolic extract (bottom) from *C. arizonica* leaves.

The aqueous and ethanolic extracts of *C. arizonica* leaves were analyzed by HPLC-PDA-MS/MS. Altogether, 67 secondary metabolites belonging to different chemical classes were annotated. The tentative identification of compounds was based on their MS and MS^2^ spectral data and retention times, as illustrated in Table 3. They included organic acids and their derivatives, phenolic acids, and their glycosides, diterpene acids, procyanidins and flavonoid glycosides such as quercetin, kaempferol and isorhamnetin-related glycosides as well as biflavonoids. Significant differences were detected from both extracts, where the aqueous extract was rich in phenolic acids and proanthocyanidins, while the ethanolic extract was rich in biflavonoids and fatty acids (Figure 4).

## 3. Discussion

Oxidative stress is considered as the main cause of skin aging through various mechanisms. Thus, the search for plants that inhibit oxidative stress-induced skin aging can be an excellent approach to delay this process. Phytochemicals such as polyphenolics are well-known for their potential antioxidant capacity. Consequently, antioxidant-synthesizing plants are promising candidates to prevent the skin-aging process. Hence, in this study, we conducted an evaluation of the in vitro antioxidant and antiaging properties of aqueous and ethanolic extracts of *C. arizonica* leaves, along with an HPLC-MS/MS analysis and assessment of their antibacterial and antibiofilm activities.

Comparatively, the aqueous extract had higher polyphenolic and flavonoid contents in addition to higher antioxidant potential than the ethanolic extract. The phytochemical profiles of both the aqueous and ethanolic extracts from *C. arizonica* leaves were investigated using the LC-MS/MS analysis, indicating that the higher antioxidant activity of the aqueous extract was most probably due to the higher contents of flavonoids, phenolic acids and proanthocyanidins predominating in the extract, which are powerful antioxidants through their free radical scavenging and metal ion chelation capacities [22]. For instance, the presence of phenolic acid derivatives remarkably enhanced the antioxidant activity, as demonstrated in many other phenolic acid-containing herbs [23]. Additionally, proanthocyanidins (monomers, dimers and trimers) found in the bark extract revealed exceptional antioxidant capacity [24]. Furthermore, the previously reported antioxidant activities of quercetin, kaempferol and isorhamnetin-related glycosides effectively confirmed their crucial role in the antioxidant activity of the extracts [25,26].

We revealed that the aqueous and ethanolic extracts from *C. arizonica* leaves are rich sources of antioxidant compounds (Table 3) that can behave synergistically against target skin-aging enzymes and accordingly improve the skin aging process. Our results are in good agreement with the reported data concerning the antiaging activity of these compounds. In particular, plants producing chlorogenic acid, catechins, and quercetin glucoside exhibited considerable in vitro antiaging activity through inhibitory effects against the previously mentioned enzymes [27,28]. Also, previous investigations on plants containing quercetin glucoside, kaempferol rutinoside and isorhamnetin glycosides demonstrated that they possess significantly anti-collagenase, anti-hyaluronidase and anti-elastase activities [29].

The Gram-negative bacterium *P. aeruginosa*, a common pathogen, is a major cause of persistent skin infections owing to its ability to form biofilms, where the bacteria are aggregated and enclosed in a self-made extracellular matrix. Most of the currently used antimicrobial agents may reduce the number of bacteria in biofilms, but they cannot completely eliminate the biofilms because of their intrinsic antibiotic tolerance and resistance, hence allowing infections to relapse [4]. Thus, the present study also aimed to confirm the antibiofilm potential of aqueous and ethanolic extracts of *C. arizonica* as well as their effects on swimming and swarming motilities of *P. aeruginosa* using two sub-inhibitory concentrations (1/4 and 1/8 MICs). The aqueous extract at the dose of 25 mg/mL (1/4 MIC) significantly inhibited biofilm production and decreased the swimming and swarming motilities of *P. aeruginosa.* This could be useful in weakening bacterial pathogenicity by destroying biofilm and reducing the spread of infections. Similar findings were described before for extracts rich in polyphenols, such as those from *Salix tetrasperma* flowers and bark [30], *Ximenia americana* leaf [30,31] and *Lavandula coronopifolia* aerial parts [32].

## 4. Material and Methods

### 4.1. Plant Material and Extraction

*C. arizonica* fresh leaves were collected from El-Orman Botanical Garden, Giza, Egypt in April 2021. A voucher sample was maintained at the botanical herbarium of Pharmacognosy Department, Faculty of Pharmacy, Zagazig University under accession number ZU-Ph-Cog-020100. The plant material was air-dried, ground, and then subjected to ultrasound-assisted extraction (UAE) using water and ethyl alcohol (10 g × 400 mL for each extraction) for 15 min under the following extraction parameters: 20 Hz, 5 °C, 30% sound wave amplification, and a pulse of 10 s. The aqueous and ethanolic extracts were filtered using glass wool, then centrifuged for 7 min at 6000 rpm. The combined filtrates (for each extract) were evaporated under reduced pressure using a Buchi rotavapor R-300 (Flawil, Switzerland), then freeze-dried to yield fine dried powders (1.14 g and 0.80 g, respectively).

### 4.2. In Vitro Assays

The antioxidant activity was estimated using two colorimetric assays, the 2,2-diphenyl-1-picrylhydrazyl (DPPH) [33] and ferric reducing antioxidant power (FRAP) assays [34], following the previously reported protocol [35]. Total polyphenolic content (TPC) and total flavonoids content (TFC) were evaluated as previously reported [35]. Detailed methods are provided in the Supplementary Materials.

### 4.3. Enzymatic Activities

#### 4.3.1. Collagenase Inhibition

The assay was conducted as previously described [27] with slight modifications using Tricine buffer (50 mM, consisting of 400 mM NaCl and 10 mM CaCl_2_, pH 7.5). Collagenase enzyme, from *Clostridium histolyticum* (ChC—EC.3.4.23.3), was dissolved in the buffer to yield an enzyme initial concentration of 0.8 unit/mL. A 2 mM solution of the synthetic substrate N-[3-(2-furyl) acryloyl] -Leu-Gly-Pro-Ala (FALGPA) was prepared by dissolving in Tricine buffer. Serial diluted samples were incubated for 15 min with the enzyme in Tricine buffer. Then, the substrate was added to each serial solution. A microplate reader was used to measure the absorbance at 490 nm. Quercetin was used as positive control. Triplicates were established for each reaction, and the collagenase inhibition (%) was calculated as follows: Inhibition (*%*) = [(*A* control − *A* sample/*A* control) × 100].

*A* sample: absorbance value of the collagenase activity from samples or positive control (the enzyme activity in presence of the extracts or positive control), *A* control: absorbance value of the collagenase activity from negative control (the enzyme activity in absence of the extracts).

#### 4.3.2. Tyrosinase Inhibition

The tyrosinase inhibition assay was carried out using L-DOPA as substrate and mushroom tyrosinase according to the previously reported method [27]. The reaction consisted of 15 μL of mushroom tyrosinase enzyme (2500 U/mL), 15 μL of samples to be tested (100–6.25 μg/mL), 100 μL of L-DOPA substrate (5 mM), and 100 μL of phosphate buffer (0.05 M, pH of 6.5). After the addition of L-DOPA, the reaction mixture was observed at 475 nm for dopachrome formation using a microplate reader. Kojic acid was used as positive control, and each reaction was performed in triplicate. The tyrosinase enzyme inhibition (%) was calculated using the following formula: Inhibition (*%*) = [(*A* control − *A* sample/*A* control) × 100]

*A* sample: absorbance value of the tyrosinase enzyme activity from samples or positive control.

*A* control: absorbance value of the tyrosinase enzyme activity from negative control (solution without samples).

#### 4.3.3. Elastase Inhibition

This assay was conducted according to a previous study [27]. A stock solution (3.33 mg/mL) of Porcine pancreatic elastase enzyme was prepared in sterile water, while a 1.6 mM solution of N-Succinyl-Ala-Ala-Ala-p-nitroanilide (AAAPVN) was prepared in buffer and used as a substrate. Test samples (100 to 6.25 μg/mL) were incubated with the enzyme for 15 min. Afterwards, AAAPVN substrate was added to initiate the enzyme reaction. The final reaction mixture had a total volume of 250 μL, consisting of 0.8 mM AAAPVN, 1 μg/mL PE, 25 μg test sample, and the buffer vehicle. A microplate reader was used to check the absorbance at 400 nm. Kojic acid was used as positive control, and each reaction was performed in triplicate. Elastase enzyme inhibition (%) was calculated as follows: Inhibition (*%*) = [(*A* control − *A* sample/*A* control) × 100]

*A* sample: absorbance value of the elastase activity from samples or positive control.

*A* control: absorbance value of the elastase activity from negative control (solution without samples).

#### 4.3.4. Hyaluronidase Inhibition

Hyaluronidase inhibition assay was performed as previously reported [27] using hyaluronidase enzyme (1.5 mg/mL) and hyaluronic acid (1 mg/mL in 0.1 M acetate buffer; pH 3.5) as a substrate. The reaction mixture contained 25 μL of 12.5 mM CaCl_2_, 12.5 μL of each test sample (100–6.25 μg/mL), 1.5 mg/mL of hyaluronidase enzyme, and 100 μL of substrate hyaluronic acid. Next, the reaction mixture was put in a water bath at 100 °C for 3 min, and then, 25 μL of KBO_2_ (0.8 M) was added to all tubes. After cooling the tubes to room temperature, 800 μL DMAB (4 g DMAB in 40 mL acetic acid and 5 mL 10 N HCl) was added to the tubes, followed by incubation for 20 min, then the contents of the tubes were transferred to respective wells in a 96-well microplate. The absorbance was read at 600 nm, and each reaction was carried out in triplicate. Kojic acid was used as a positive control. Hyaluronidase enzyme inhibition (%) was calculated as follows: Inhibition (*%*) = [(*A* control − *A* sample/*A* control) × 100]

*A* sample: absorbance value of the hyaluronidase activity from samples or positive control.

*A* control: absorbance value of the hyaluronidase activity from negative control (solution without samples).

### 4.4. Antibacterial Activities

#### 4.4.1. Determination of the Minimum Inhibitory Concentration (MIC)

The MIC of *C. arizonica* aqueous and ethanolic leaf extracts against *Pseudomonas aeruginosa* was determined by the broth microdilution assay in a 96-well microtiter microplate [36,37]. First, the samples were dissolved in Muller–Hinton (MH) broth with 5% of dimethyl sulfoxide (DMSO) to obtain a final concentration of 100 mg/mL. Next, the extracts were sterilized using 0.22 μm sterile syringe filters and two-fold serially diluted into the plate’s wells in triplicate with final extract concentrations ranging from 1.562 to 100 mg/mL. After completing the volume to 200 μL per well by MH medium, fresh overnight suspensions of *P. aeruginosa* adjusted to a turbidity of OD_600nm_ = 0.6 were introduced into each well (2 μL per well). Wells with no bacterial inoculation were established as negative controls. In addition, media without extract amendment were used as growth controls, and ampicillin (10–0.078125 mg/mL) was established as the reference antibiotic. The plate was incubated at 37 °C under shaking at 150 rpm under aerobic conditions. After 18 h incubation, the bacterial growth was visually and spectrophotometrically checked. The lowest concentration of the extract that inhibited the visible growth of the bacterium was defined as the MIC.

#### 4.4.2. Biofilm Inhibition Using Crystal Violet Assay

The effects of the sub-MIC concentrations of *C. arizonica* aqueous and ethanolic leaf extracts on biofilm formation by *P. aeruginosa* were determined using the crystal violet colorimetric assay [30,31]. Briefly, the extracts at the doses of 1/8 MIC and 1/4 MIC were prepared in MH broth, filtered using 0.22 μm syringe filters, inoculated, and incubated as described above in the MIC assay. After incubation, the bacterial suspensions were discarded, and the wells were gently washed thrice with a phosphate-buffered saline (PBS) solution to remove nonadherent bacterial cells. Thereafter, adherent bacteria were stained by a 1% crystal violet (CV) solution (200 μL per well) and incubated for 15 min at room temperature. Next, CV solutions were discarded, and wells were washed with sterile distilled water to remove excess dye. The plate was air-dried, and the attached biofilm was solubilized by adding 200 μL of 95% ethanol into each well. The amount of biofilm was estimated spectrophotometrically at OD_600nm_ using a multimode plate reader. Media without the extracts’ supplementation were used as positive controls for biofilm production.

#### 4.4.3. Swimming and Swarming Motility Assessments on Plates

*C. arizonica* aqueous and ethanolic leaf extracts at 1/4 and 1/8 sub-MIC concentrations was evaluated on the swimming and swarming motilities of *P. aeruginosa* [30]. The swimming (0.5% NaCl, 1% tryptone, and 0.3% agar) and swarming (LB medium, 0.6% agar) plate media [38] were prepared and autoclaved. When the media were relatively cool (<50 °C), they were supplemented with the extract filtered beforehand to obtain final doses of 1/8 and 1/4 MIC. The plates were left to dry under a laminar flow hood. Next, 10 μL of a fresh suspension of *P. aeruginosa* adjusted to OD_600nm_ = 0.6 were deposited at the middle of agar plates and incubated at 37 °C for 24 h. Media without extracts were established as controls. The motility zone diameters were measured in cm.

### 4.5. HPLC-PDA-MS/MS Analysis

The phytochemical analysis was performed using HPLC-PDA-MS/MS. A SHIMADZU LC MS 8050 (Shimadzu, Japan) LC system was utilized alongside a triple quadruple spectrometer with an ESI source. A C18 reversed-phase column (Zorbax Eclipse XDB-C18, rapid resolution, 4.6 × 150 mm, 3.5 µm, Agilent, Santa Clara, CA, USA) was used for the separation process. Water and acetonitrile (ACN) (0.1% formic acid each) gradients were employed from 5 % to 30 % ACN over 60 min with a flow rate of 1 mL/min. The injection of samples was automatically performed using autosampler SIL-40C xs. LC solution software (Shimadzu, Japan) was used to operate the instrument. The MS operated in the negative mode.

### 4.6. Statistical Analysis

Each experiment was carried out three times throughout the study, and the results were provided as means ± standard deviation (SD). The Tukey’s post hoc test was performed to process for significant differences between the group means using IBM SPSS software. It was deemed statistically significant when *p* ˂ 0.05.

## 5. Conclusions

In this study, the phytochemical characterization of the aqueous and ethanolic leaf extracts of *C. arizonica* revealed 67 secondary metabolites belonging mainly to flavonoids, biflavonoids, phenolic acids and proanthocyanins. The aqueous extract displayed substantial in vitro antioxidant activity and appreciable inhibitory activity against the four crucial enzymes (collagenase, elastase, tyrosinase, and hyaluronidase) involved in skin aging. Moreover, the extract exhibited promising antibiofilm activity against *P. aeruginosa* and impaired its motility. To summarize, this wide range of activities (antioxidant and antibacterial activities) of *C. arizonica* extracts could be due to their high polyphenolic contents, which confer their promising dermato-cosmeceutical properties. Therefore, *C. arizonica* leaf extract could be regarded as a prospective candidate for further investigation as an agent to be utilized against skin aging and its associated symptoms.

## Figures and Tables

**Table 1:** In vitro antioxidant activities (DPPH, FRAP) and TPC and TFC for the aqueous and ethanolic extracts of *C. arizonica* leaves.

Plant Organ	Extract	DPPH	FRAP	TPC	TFC
		IC_50_, µg/mL	mM of FeSO_4_/g Extract	mg GA/g Extract	mg QE/g Extract
Leaf	Aqueous	55.45 ± 0.51	15.1 ± 0.04	139.99 ± 0.04	1.54 ± 0.05
	Ethanolic	61.89 ± 0.11	11.56 ± 0.01	88.86 ± 0.08	1.12 ± 0.16
Reference compound	Quercetin	0.23 ± 0.01	24.04 ± 1.23	-	-
	BHT	4.21 ± 0.08	-	-	-

BHT: Butylated hydroxytoluene; TPC: Total polyphenols content; TFC: Total flavonoids content.

**Table 2:** In vitro enzyme inhibitory activities of the aqueous and ethanolic extracts of *C. arizonica* leaves.

Plant Organ	Extract	Parameters IC_50_ (µg/mL)			
		Elastase	Tyrosinase	Collagenase	Hyaluronidase
Leaf	Aqueous	37.36 ± 4.01	32.45 ± 5.1	41.5 ± 1.1	38.15 ± 0.1
	Ethanolic	50.01 ± 1.24	54.4 ± 6.34	45.6 ± 3.23	52.6 ± 2.45
Reference compound	Kojic acid	21.60 ± 0.9	9.00 ± 0.9	-	14.46 ± 0.6
	Quercetin	-	-	24.83 ± 1.8	-

**Table 3:** Annotated metabolites by HPLC-MS/MS analyses of aqueous and ethanolic extracts from *C. arizonica* leaves.

No.	Rt	M-H	MS/MS	Proposed compounds	Extracts	
					Aqueous	Ethanol
1.	1.64	191	146	Quinic acid	++	++
2.	1.68	133	114	Malic acid	++	++
3.	1.98	191	111	Citric acid	++	+
4.	2.01	289	111	Quinic acid malate	++	+
5.	2.67	237	115	Benzoyl malic acid	++	+
6.	3.65	609	305	(epi)Gallocatechin-(epi)gallocatechin	++	+
7.	3.66	331	169	Galloyl glucose	++	+
8.	4.20	609	305	(epi)Gallocatechin-(epi)gallocatechin	++	+
9.	4.59	865	289	Proanthocyanidin trimer	++	+
10.	4.86	593	289	(epi)Catechin-(epi)gallocatechin	++	+
11.	4.92	315	153	Dihydroxybenzoic acid glucoside	++	+
12.	5.10	329	167	Vanillic acid glucoside	++	+
13.	5.73	305	179	(epi)Gallocatechin	++	+
14.	5.83	897	289	(epi)catechin-*O*-gallate-(epi)gallocatechin-*O*-gallate	++	+
15.	5.85	345	169	Gallic acid glucuronide	++	+
16.	6.25	315	153	Dihydroxybenzoic acid glucoside	++	+
17.	6.67	593	305	(epi)Catechin-(epi)gallocatechin	++	+
18.	7.21	593	289	(epi)Catechin-(epi)gallocatechin	++	+
19.	7.66	325	163	Hydroxycinnamic acid glucoside	++	+
20.	7.18	897	289	(epi)Catechin gallate-(epi)gallocatechin-*O*-gallate	++	+
21.	8.47	447	169	Gallic acid glucosyl malate	++	+
22.	8.50	881	289	(epi)Catechin-*O*-gallate-(epi)catechin-*O*-gallate	++	+
23.	8.86	337	191	Coumaroylquinic acid	+	+
24.	9.19	577	289	(epi)Catechin-(epi)catechin	++	+
25.	9.37	593	289	(epi)Catechin-(epi)gallocatechin	++	+
26.	10.03	337	191	Coumaroylquinic acid	++	+
27.	10.84	881	289	(epi)Catechin-*O*-gallate-(epi)catechin-*O*-gallate	++	+
28.	11.20	289	245	(epi)Catechin	++	+
29.	12.04	865	289	Proanthocyanidin trimer	++	+
30.	12.86	577	289	(epi)Catechin-(epi)catechin	++	+
31.	14.42	431	153	Dihydroxybenzoic acid derivative	++	++
32.	15.75	337	191	Coumaroylquinic acid	++	+
33.	16.83	341	179	Caffeoyl glucose	++	+
34.	17.37	327	165	Phloretic acid glucoside	++	+
35.	18.48	507	153	Dihydroxybenzoic acid derivative	++	+
36.	18.87	577	289	(epi)Catechin-(epi)catechin	++	+
37.	19.90	625	317	Myricetin rutinoside	++	+
38.	20.13	507	269	Apigenin derivative	+	+
39.	21.22	523	361	Acetoxyisocupressic acid glucoside	++	+
40.	22.36	345	165	Phloretic acid glucoside	++	+
41.	21.76	521	359	Caffeoyl rosmarinic acid	+	+
42.	23.61	609	301	Rutin	++	+
43.	24.04	463	301	Quercetin glucoside	++	+
44.	24.25	593	285	Kaempferol rutinoside	++	+
45.	24.37	551	315	Isorhamnetin glyceryl glucoside	+	+
46.	24.67	463	301	Quercetin glucoside	++	+
47.	25.54	523	361	Acetoxyisocupressic acid glucoside	++	+
48.	27.07	593	285	Kaempferol pentosyl glucoside	++	+
49.	28.37	447	285	Kaempferol glucoside	++	+
50.	28.49	699	315	Isorhamnetin derivative	++	+
51.	29.21	477	315	Isorhamnetin glucoside	++	+
52.	30.26	461	315	Isorhamnetin rhamnoside	++	+
53.	49.07	537	375	Biapigenin	+	++
54.	49.36	553	391	Dihydro-biapigenin methyl ether	+	++
55.	50.51	553	391	Dihydro-biapigenin methyl ether	+	++
56.	50.69	537	375	Biapigenin	+	++
57.	51.41	565	223	Isoginkgetin	+	++
58.	51.65	537	375	Biapigenin	+	++
59.	51.99	553	391	Dihydro-biapigenin methyl ether	+	++
60.	52.68	539	295	Chrysocauloflavone I	+	+
61.	54.87	551	375	Bilobetin		++
62.	55.92	537	375	Biapigenin	+	++
63.	56.10	553	377	Dihydroisocryptomerin	+	++
64.	59.76	551	283	Methyl-amentoflavone		++
65.	62.35	579	335	Trimethylamentoflavone	+	++
66.	65.46	379	253	Octanedioic acid		++
67.	67.40	311	183	Dioxooctadecanoic acid	+	++

+: minor; ++: major

## Data Availability

The data presented in this study is contained within the article or Supplementary Material.

## Supplementary Materials

The following supporting information can be downloaded at: https://www.mdpi.com/article/10.3390/molecules28031036/s1.

## Sample Availability

Samples of the compounds are not available from the authors.
