# Supplementary material for: Cupressus arizonica Greene: Phytochemical Profile and Cosmeceutical and Dermatological Properties of Its Leaf Extracts
Source: Molecules. 2023 Jan 20;28(3):1036. doi: 10.3390/molecules28031036 (PMC9919735; doi:10.3390/molecules28031036)
Supplement: Supplementary file 1 [file molecules-28-01036-s001.zip › molecules-2088923-supplementary.pdf]

molecules

Supplementary Materials

Cupressus arizonica Greene: Phytochemical Profile and Cosmeceutical and Dermatological Properties of Its Leaf Extracts

Nora Tawfeek [1,*], Eman Fikry [1], Ismail Mahdi [2], Melvin Adhiambo Ochieng [2], Widad Ben Bakrim [2,3], Noamane Taarji [2], Mona F. Mahmoud [4] and Mansour Sobeh [2,*]

[1] Department of Pharmacognosy, Faculty of Pharmacy, Zagazig University, Zagazig 44519, Egypt; efhassan@zu.edu.eg
[2] AgroBioSciences, Mohammed VI Polytechnic University, Lot 660–Hay MoulayRachid, Ben-Guerir 43150, Morocco; ismail.mahdi@um6p.ma (I.M.); melvin.ochieng@um6p.ma (M.A.O.); widad.benbakrim@um6p.ma (W.B.B.); noamane.taarji@um6p.ma (N.T.)
[3] African Sustainable Agriculture Research Institute (ASARI), Mohammed VI Polytechnic University (UM6P), Laayoune 70022, Morocco
[4] Department of Pharmacology and Toxicology, Faculty of Pharmacy, Zagazig University, Zagazig 44519, Egypt; mfabdelaziz@zu.edu.eg
* Correspondence: noratawfeek1@gmail.com (N.T.); mansour.sobeh@um6p.ma (M.S.)

Methods

4.2.1. 2,2-diphenyl-1-picrylhydrazyl (DPPH) and ferric reducing antioxidant power (FRAP) assays

The antioxidant activity was estimated using two colorimetric assays, DPPH [33] and FRAP assays [34], following the previously reported protocol [35]. In the DPPH assay, 100 µL of DPPH solution (200 µM) was added to 100 µL of each extract (with concentrations ranging between 500 and 1.25 µg/mL). The samples were incubated for 30 min in the dark at room temperature. The absorbance was observed at λ max 517 nm using a microplate reader. The ability of the samples to scavenge the DPPH radicals was calculated according to the following equation:

DPPH scavenging effect (%) = [(A0 –A1)/A0] ×100

where A0 is the absorbance value of the control, and A1 is the absorbance value in the presence of the extracts. All measurements were carried out in triplicate. The IC_{50} value (the concentration that exhibited 50% scavenging for DPPH radicals) was expressed as µg/mL and evaluated by sigmoid non-linear regression using adequate software.

In the FRAP assay, 180 µL of freshly prepared warm FRAP working solution was added to the samples (20 µl). The reaction was kept for 7 min at 37 °C. All measurements were performed in triplicate. The reduction was noticed by the appearance of an intense blue coloration, which was measured at 595 nm using a spectrophotometer microplate reader Tecan Safire IITM spectrophotometer. FRAP values were expressed as mM $FeSO_4$/mg sample.

4.2.2. Total polyphenolic content (TPC) and total flavonoids content (TFC)

TPC and TFC were measured as previously reported [35].

TPC was evaluated using the Folin–Ciocalteu method for both extracts, where 20 µL of the sample solution (5 mg/mL) were mixed with different serial dilutions of the standard gallic acid (with concentrations ranging from 50 to 1 µg/mL) in a 96-well microplate. Then, Folin–Ciocalteu's reagent (100 µL) was added to the wells, keeping the samples for 5 min at room temperature. A 7.5% solution of sodium carbonate solution (80 µL) was

added, then the plate was mixed and kept in dark at room temperature for 2 h. Finally, the absorption was measured at 750 nm using a microplate reader. The calibration curve was plotted using different serial dilutions of the standard compound, gallic acid. The phenolics contents of both extracts were expressed in terms of gallic acid equivalent (GAE)/g plant extract.

TFC was evaluated using the aluminum colorimetric technique, where 150 μL of 0.4 mg/mL of both extracts were mixed with 100 μL of 2% (w/w) $AlCl_3$ in a 96-well microplate, incubated at 37 °C for 30 min, and read at λ max 415 nm with a microplate reader against a blank without aluminum chloride. Quercetin was employed as the standard, and the calibration curve was plotted versus standard curve of quercetin. The data were shown as quercetin equivalent (QE) per g of extract.
